# Understanding the molecular basis of substrate binding specificity of PTB domains

**DOI:** 10.1038/srep31418

**Published:** 2016-08-16

**Authors:** Neetu Sain, Garima Tiwari, Debasisa Mohanty

**Affiliations:** 1 Bioinformatics Center, National Institute of Immunology, Aruna Asaf Ali Marg, New Delhi–110067, India

## Abstract

Protein-protein interactions mediated by phosphotyrosine binding (PTB) domains play a crucial role in various cellular processes. In order to understand the structural basis of substrate recognition by PTB domains, multiple explicit solvent atomistic simulations of 100ns duration have been carried out on 6 PTB-peptide complexes with known binding affinities. MM/PBSA binding energy values calculated from these MD trajectories and residue based statistical pair potential score show good correlation with the experimental dissociation constants. Our analysis also shows that the modeled structures of PTB domains can be used to develop less compute intensive residue level statistical pair potential based approaches for predicting interaction partners of PTB domains.

PTB domain (Phosphotyrosine binding domain) containing proteins modulate a wide range of physiological processes including neuronal development, immune responses, tissue homeostasis and cell growth^1^,^2^,^3^. Since PTB domains are involved in signaling networks as critical scaffold/adaptor proteins, a better understanding of PTB mediated interactions will help in therapeutic intervention for specific diseases. The PTB domains typically consist of 100 to 170 amino acid residues and a number of crystal structures have been elucidated for PTB domains from various organisms. The core of the domain consists of seven anti parallel β strands forming two orthogonal β sheets, capped at the C-terminus by α helix (Fig. 1). PTB domain basically binds to phosphotyrosine (pY) containing peptides having the sequence motif NPXpY/NXXpY that adopt a beta-turn providing an anchor point for binding. Apart from PTB domains, which recognize phosphorylated tyrosine containing peptides, a subfamily of PTB domains can also recognize peptides having unphosphorylated tyrosines. The PTB domain family can be divided into three subgroups on the basis of structure/function relationships, namely, IRS-like, Shc-like and Dab-like^4^. Based on structure based sequence alignments, it has been proposed that large evolutionary divergence exits between the IRS-like and Shc/Dab-like PTBs^4^. In an effort to better understand the structural basis of binding specificity of PTB, both X-ray crystallography and NMR solution structures of PTB domain in complex with peptides have been elucidated by several groups^5^,^6^,^7^,^8^,^9^,^10^. Apart from solving structures, mutagenesis studies and binding assays have also been used to determine the specificity of PTB domains towards their interacting partners. Smith *et al*. have used an *in vitro* method (peptide SPOTS array) to screen a number of sequences derived from receptors in the human proteome for PTB domain ligands^11^. This study has highlighted the importance of amino acids flanking the NXXY motif as determinants of specificity. But the studies focused only on very few PTB-protein pairs.

Although there is lack of high throughput data for deciphering the specificity of PTB domain mediated interactions, availability of X-ray and NMR structures of PTB domains belonging to different families, opens up the opportunity for understanding the structural basis of substrate recognition by PTB domains. Molecular dynamics simulations have been used previously to assess the role of tyrosine phosphorylation of Shc protein in regulating the binding affinity of SH2 and PTB domains for its interaction partners^12^. Recently available high throughput experimental data on PTB-peptide interactions suggest that, even though NPXY, NPXpY or NXXY motifs are necessary for recognition of substrates by PTB domains, sequences containing identical motifs show differential specificity for different PTB domains. This highlights the role of sequences flanking PTB domains and potential limitations of motif based approaches in prediction of substrates of PTB domains. The structure based approaches for prediction of protein-peptide interactions can decipher the role of flanking residues by modeling potential substrates in PTB binding pockets and ranking them as per their binding energy by using suitable scoring functions.

Molecular dynamics (MD) simulations provide structural details at atomic resolution and computational methods like Molecular Mechanics/Poisson-Boltzmann Solvent Area (MM/PBSA)^13^ approach facilitate *in silico* evaluation of binding energy. However, because of compute intensive nature of MD simulations it is not practical to use MD and MM/PBSA as a tool for searching potential interaction partners of PTB domains, which would require simulations of both binder and large numbers of non-binder peptides in complex with every PTB domain. Therefore, it is necessary to develop computational methods which utilize structural information but are less compute intensive. Use of residue based statistical pair potentials facilitate fast screening of large number of putative substrate peptides for a given peptide recognition module (PRM) like PTB domain. In earlier work from our laboratory, we have analyzed the performance of structure based approach involving statistical pair potentials for predicting binding partners of MHCs, kinases and PDZ domains^14^,^15^,^16^. The major objective of the current manuscript was to investigate if similar structure based approach can be applied for predicting binding partners of PTB domains.

In this work, we have carried out detailed analysis of the sequence and structural features of the available free as well as substrate bound PTB domains in PDB to identify suitable structural templates for modeling PTB domains belonging to different families. For a representative PTB domain, a set of peptides with known binding affinities have been modeled in the PTB binding pocket and detailed all atom molecular dynamics (MD) simulations have been carried out to identify their binding modes taking into account the conformational flexibilities of the PTB-peptide complexes. Binding free energies for these PTB-peptide complexes have been computed from the MD trajectories by MM/PBSA approach. In order to benchmark the ability of the all atom computational approach to predict the effect of residues flanking the NPXY motif on binding energies of the peptides, the predicted MM/PBSA binding free energies have been compared with the experimentally determined binding affinities. MD simulations on PTB-peptide complexes have also helped in identifying the crucial binding pocket residues which play key role in peptide recognition. Hence, using these binding pocket residues obtained from analysis of MD trajectories, it is also possible to use less compute intensive residue based statistical pair potentials and obtain computational binding energies in good correlation with experimental values. Next, attempt has been made to investigate whether the static crystal structures of PTB domains can be used directly in combination with knowledge based scoring functions like residue based statistical pair potentials for developing fast and efficient models for scoring PTB-peptide complexes.

## Results

### Analysis of the three dimensional structures of PTB domains

Protein Data Bank (PDB)^17^ was scanned to retrieve the structures of PTB domain which were then classified into two groups-first peptide bound and second unbound. A total of 34 peptide bound structures were selected for study and they consisted of 20 X-ray and 14 NMR structures. The total number of unbound structures considered for this analysis was 27, out of which 17 were X-ray and 10 were NMR structures. Out of these 61 structures, both with and without peptide, a set of 39 non-redundant entries were selected for further analysis. The criteria for retrieval of these 61 structures and selection of 39 structures out of them, have been described in the methods section. Figure 2 shows the sequence based phylogenetic tree of a non-redundant set of 39 PTB domains belonging to these seven clusters and the structural similarities between them as measured by RMSD values obtained using TM-align^18^ are given in Supplementary Figure S1. In order to investigate the sequence and structural differences between the clusters, representative structures were selected from each of these clusters and an all-against-all sequence comparison was carried out using pairwise BLAST and structure comparisons were carried out using TM-align program^18^. Supplementary Figure S2 shows the results of sequence and structural comparison of representative PTB-peptide complexes selected from each of the seven clusters. All of these except 1PFJ are in complex with peptide. In all of the cases, pairwise sequence identity between members of two different clusters was below 27.66% and often they do not show statistically significant sequence similarity in BLAST alignment. However, despite the high divergence in sequence, the structures align with RMSDs in the range of 1.8 to 2.94 Å over 91–134 residues *i.e.* over 90% of the length of the PTB domains except 3VRP which is a SH2-like domain. These results suggest that PTB domains have a conserved structural fold despite high sequence divergence.

### Dynamic behavior of PTB-peptide complexes and calculation of MM/PBSA binding free energy

**Figure 1:**
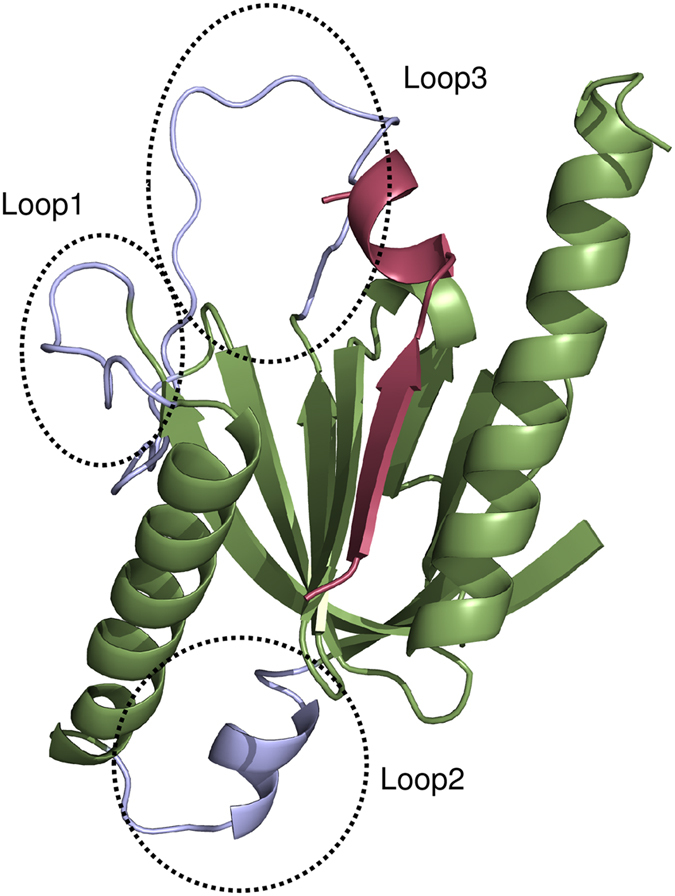
X11 PTB domain structure. Cartoon representation of structure of X11 PTB domain in complex with 13mer peptide (PDBID: 1X11). The PTB domain is colored light green and 13mer peptide is colored red. The missing residues in the three loop regions of the crystal structure have been modeled using SWISS-MODEL server and are colored light blue.

**Figure 2:**
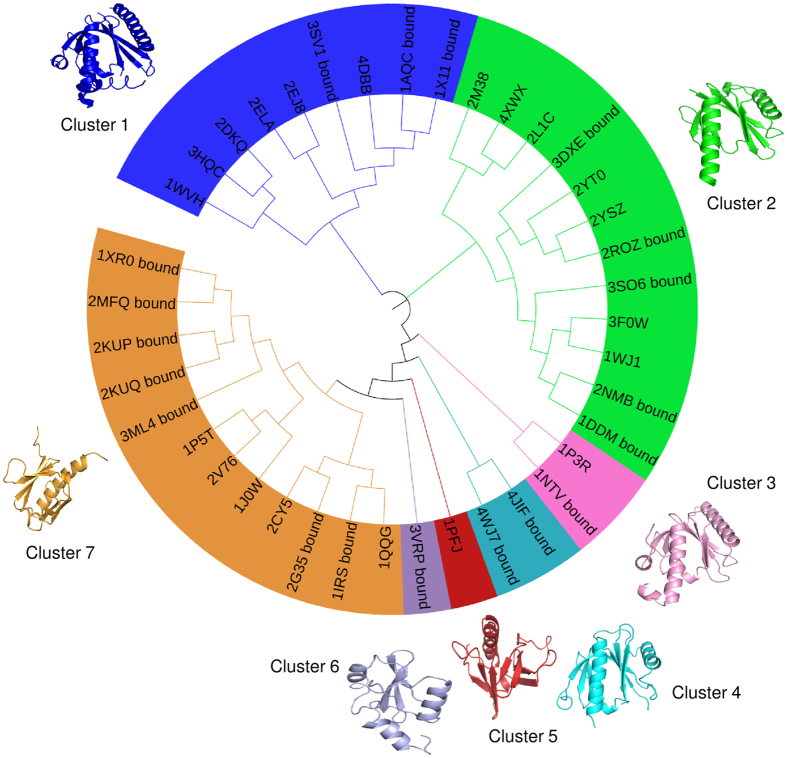
Dendrogram of PTB domains. Sequence based dendrogram for clustering of a non-redundant set of 39 PTB domains (both without peptide and in complex with the peptide) retrieved from PDB. Representative structures from each cluster are also shown.

**Figure 3:**
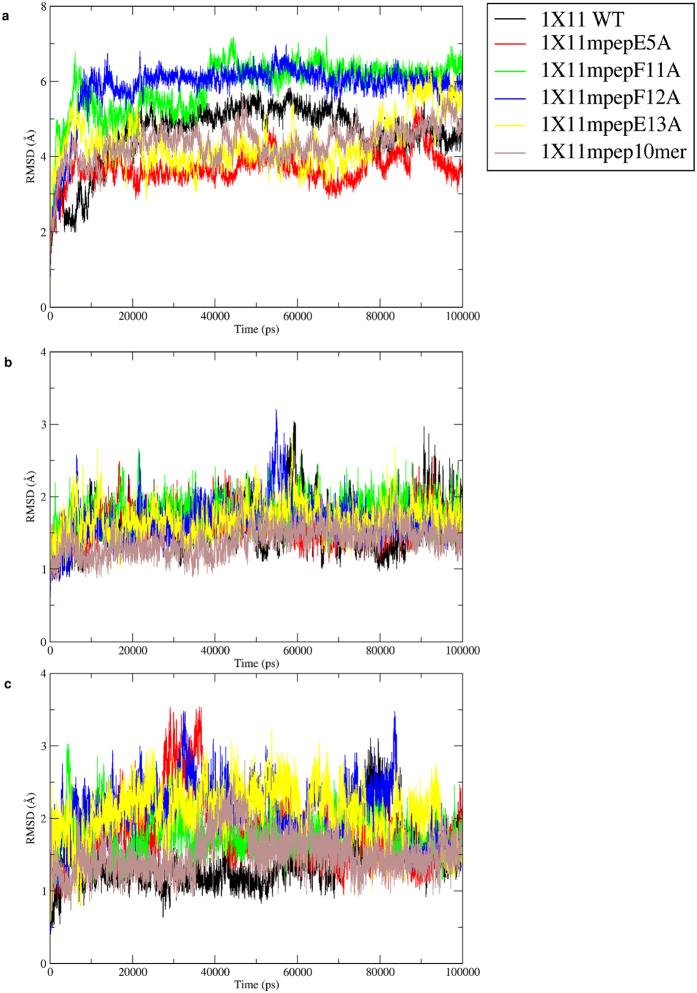
RMSD plot of X11 PTB domain and its mutants. (**A**) RMSD *vs* Time plot for the 100 ns MD simulation on PTB domain of X11 protein in complex with its cognate peptide (PDB ID: 1X11) and the mutated peptides. The inset shows color coding for different simulations. (**B**) RMSD *vs* Time plot for the 100 ns MD simulation on PTB domain of X11 protein in complex with its cognate and mutated peptides. RMSD has been calculated excluding the loop regions (represented as dotted line in Fig. 1). (**C**) RMSD *vs* Time plot for the peptides only obtained from the 100 ns MD simulation on PTB domain of X11 protein in complex with its cognate and mutated peptides.

In order to understand the role of residues flanking the NPXY motif on binding affinity, a set of peptides with known binding affinities were modeled in complex with the PTB domain using all atom explicit solvent MD simulations and binding energies were computed from simulations. The PDB entry 1X11 corresponding to PTB domain from X11 in complex with the APP peptide was chosen for this purpose. The experimental binding affinity of APP and a set of mutant peptides for X11 PTB domain has been reported by Zhang *et al*.^19^. Supplementary Table S1 summarizes the sequence of the peptides and duration of MD simulations carried out on each PTB-peptide complex. Figure 3A shows the variation of the backbone RMSD of PTB domain with respect to the starting structure for PTB-peptide complex involving the native and mutated peptides. As can be seen for the cognate peptide, RMSD values converged to 4.5 Å and it is going up to 6 Å for mutated complexes. It may be noted that, the structure contains loop regions (circled regions in Fig. 1) for which coordinates were absent in the crystal structure presumably because of its higher flexibilities and while preparing the initial structure for the simulation the said loop regions were modeled. Hence, the RMSD values were also calculated excluding the loop regions. RMSD analysis of all six PTB-peptide complexes confirmed that higher RMSD values were arising from larger movement of loop regions and after exclusion of the loop regions, the RMSD values with respect to the starting structure remained within 1.5 Å to 3.0 Å (Fig. 3B). In order to analyze the movement of the peptide during the simulation, RMSD values for the bound peptides alone with respect to their initial structures were also computed over the simulation trajectories (Fig. 3C). As can be seen, the peptide RMSDs remained within 2 Å in all six MD simulations for cognate and mutant peptides in complex with PTB domains. It also confirms that peptides remained within the binding pocket throughout the MD simulation.

We also performed 100 ns simulation on ligand free structure of 1X11 to compare the conformational flexibilities of the loop region in the peptide bound and unbound PTB domain. Supplementary Figure S3 shows the comparison between RMSDs of the loop regions over the 100 ns trajectory in peptide free and peptide bound structures of 1X11. As can be seen in absence of the bound peptide the RMSD of the loop region shows large fluctuations over the 100 ns trajectory. This higher flexibility might be the reason for the lack of coordinates of these loops in the crystal structure. On the other hand in presence of the bound peptide, the loop1 and loop3 deviate significantly with respect to their initial modeled conformation, but after 20 ns they stabilize to a conformation which is optimal for peptide binding and show much lower fluctuations in RMSD compared to unbound 1X11 simulation. Thus our simulations essentially demonstrate that the flexible loop regions become more ordered upon peptide binding and loop flexibility plays an important role in formation of suitable peptide binding site.

We wanted to investigate whether the binding free energy values computed by our simulations correlate with the experimentally determined binding affinity values for these six PTB binding peptides. For each complex MM/PBSA binding free energy was calculated from the last 20 ns of the 100 ns trajectory. Supplementary Figure S4 and Table 1 shows the binding energy values calculated by MM/PBSA approach, experimental K_d_ values and experimental binding free energy obtained from K_d_ values^19^. Interestingly, the trend shown by computational binding free energy values are similar to the experimental binding free energy values. Table 1 also shows the correlation coefficient between the computational and experimental binding energy values for these six PTB-peptide complexes. It is encouraging to note that the correlation coefficient is 0.63, which indicates that our simulations have successfully captured the effect of the mutations on the PTB binding affinity with reasonable accuracy.

### Interactions between the PTB domain and NPXY motif containing peptide

**Figure 4:**
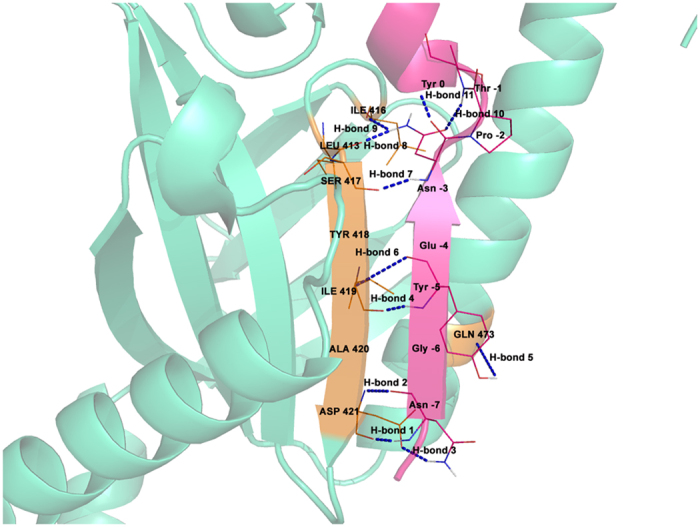
Hydrogen bonds in X11 protein-peptide complex. Schematic representation of hydrogen bonding interactions between PTB domain of X11 protein and its cognate 13mer APP peptide. Different hydrogen bonds are numbered.

As already reported in literature, peptides having high affinity towards PTB domains mostly contain NPXY motif which adopts a β-turn conformation^20^. Structural analysis of the PTB-peptide complexes has already shown that the peptide fits into the groove on the peptide-binding surface of the PTB domain. The floor of the groove is formed by strand β5, which is engaged in anti-parallel hydrogen bonding interactions between peptide residues numbered from −7 to −3. Figure 4 shows the pictorial representation of various interactions between the APP peptide and X11 PTB domain in the crystal structure^19^. A total of 11 hydrogen bonds (Fig. 4) are formed between PTB domain and peptide involving anti-parallel backbone hydrogen bonds as well as interactions involving side chains. Therefore, MD trajectories for all six PTB-peptide complexes were analyzed to check if these 11 hydrogen bonds remained stable during the MD simulations. Hence, hydrogen bond distances were calculated from the trajectories generated by molecular dynamics simulations. This analysis was done for trajectories of all six PTB-peptide complexes. Hydrogen bonds 1 and 2 between Asn (−7) and Asp 421 broke during the simulations and the interacting atom pairs moved as far as 8 Å in most of the complexes. Similarly hydrogen bond involving side chains of Asn (−7) and Asp 421 also did not remain stable during the simulations (Supplementary Figure S5a). In contrast, hydrogen bonds 4 and 6 between Tyr (−5) and Ile 419 remained quite stable throughout the simulation maintaining a distance of 3.0 Å (Supplementary Figure S5b). Hydrogen bond 5 between Tyr (−5) and Gln 473 was highly flexible and at the end of the simulation, the corresponding distance was going upto 14 Å. Asn (−3) residue of the NPXY motif plays an important role in terms of stabilizing the β-turn conformation and thus an important determinant for interaction with the protein. Left panel of Supplementary Figure S5c shows the hydrogen bond distance calculated over the simulation length between –NH of Asn (−3) and carbonyl of Ser 417, which is found to be approximately 3.0 Å in all six cases. As depicted in Fig. 4 the amino group of the side chain of Asn (−3) is involved in hydrogen bonding interactions with carbonyl groups of Leu413 and Ile416 (hydrogen bonds 8 & 9). As can be seen from Supplementary Figure S5c the hydrogen bonds 8 and 9 also remain stable during the simulation. Similarly intra peptide hydrogen bonds 10 and 11 in the NPTY region also remained in the range of 3 to 4.5 Å throughout the simulation (Supplementary Figure S5d). These stable backbone-backbone hydrogen bonds confirm that the peptides remain within the binding pocket of PTB domain and form strong hydrogen bonding network.

Apart from hydrogen bonding interactions, aromatic-aromatic interactions^21^ between peptide and PTB domain residues also play an important role in recognition and binding of the peptides with PTB domains. Supplementary Table S2 also lists all the residues of the PTB domain, which are in contact with the residues in the substrate peptides and nature of contacts (protocol described in methods). As can be seen, C-terminal residues *i.e*. the two Phe residues at +2 and +3 position have favorable contributions towards the binding affinity through aromatic-aromatic interactions. They are also in close proximity to the other aromatic amino acids like Phe and Tyr. Pro at −2 position also contributes favorably to the binding energy because of its interactions with Phe and Tyr. It must be clarified that, we have modeled various mutant peptides using crystal structure of 1X11 PTB domain in complex with the native peptide and mutant peptides have same backbone conformation as the native peptide. Major assumption in our study is that, during the simulation peptide can sample various conformations and various conformations of the side chains of the binding pocket residues of the PTB domain will also be sampled during the simulation. The system will converge to an energetically favorable binding configuration at the end of the simulation. By incorporating the flexibility into the PTB-peptide complex, we were able to obtain additional contacts which were not present in the static crystal structure 1X11, because energetically favorable interactions were obtained by conformational rearrangement of side chains. We computed the MM/PBSA binding energy by averaging over the conformations of PTB-peptide complexes sampled during last 20 ns of the 100 ns simulation. The observed correlation between experimental and MM/PBSA energy suggest that our simulations are able to reproduce the substrate recognition process to a large extent and hence SDRs identified by our simulation might be correct. Using the same set of specificity determining residues (SDRs) obtained from MD simulation, binding energy computed by residue based statistical pair potential also shows good correlation with experimental binding energy. This further supports our assumption about identification of SDRs. Thus, our simulations have probably identified the crucial specificity determining residues (SDRs) of PTB domains taking into account the conformational flexibility of the PTB-peptide complex.

### Identification of binding partners of PTB domains by statistical pair potential

MD and MM/PBSA being extremely compute intensive processes they cannot be easily used for genome scale search of potential PTB binding partners. Therefore, it is necessary to investigate alternate scoring functions, which can help in predicting PTB binding partners with reasonable accuracy. Earlier studies on protein-peptide interactions involving various PRMs like kinases, MHCs and PDZ domains have demonstrated that statistical pair potentials like Miyazawa and Jernigan (MJ)^22^,^23^ and Betancourt and Thirumalai (BT)^24^ scoring matrices can predict binding partners of PRMs with reasonable accuracy, provided binding pocket residues are correctly identified^14^,^15^,^25^. Since, we have simulated and refined the 1X11 PTB-peptide structures containing native and mutated peptides, we identified peptide binding pocket residues from the final structure obtained from our 100 ns simulation and investigated whether statistical pair potentials can rank the six PTB binding peptides as per their binding affinities. Table 1 summarizes the experimentally determined K_d_ values, binding energy values calculated by MM/PBSA and the pair potential scores calculated by BT matrix for all six PTB-peptide complexes. Interestingly, the binding energy scores calculated using BT matrix showed a correlation coefficient of 0.85 (p-value: 0.03) with experimental binding free energies. These results indicate that pair potentials are able to compute binding energy scores with reasonable accuracy based on SDRs identified from MD simulated structures. Supplementary Table S3 lists the residues of the PTB domain, which are in contact with the mutated residues in the substrate peptides as compared to native peptide and their BT score is written in parentheses (protocol described in methods). It clearly shows when fifth glutamate of peptide is mutated to alanine, salt bridge between E (−4) and R 353 is disrupted which was contributing towards the affinity. Similarly when phenylalanine is replaced with alanine, an extra arginine makes the score less negative i.e., hinder its favorable interactions in both the cases of phenylalanine mutations i.e., F11A and F12A. The last glutamate mutation to alanine resulted in disruption of three favorable interactions and when alanine is present in place of this, positive BT score is obtained. This in general explains why mutated peptides have less affinity than native peptide for 1X11 PTB domain.

### Benchmarking of the predictive power of statistical pair potential on a larger data set of PTB binding peptides

**Figure 5:**
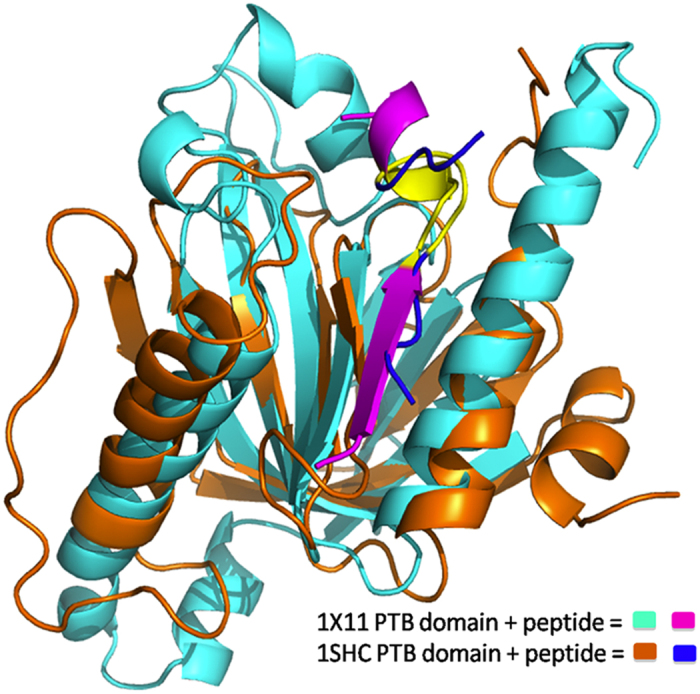
Superimposition of 1X11 and 1SHC. Structural superimposition of 1X11 and 1SHC in complex with their cognate peptides. NPxY motif in both the peptides has been highlighted in yellow color.

Since the binding energy score calculated using Betancourt and Thirumalai (BT) statistical pair potential was in agreement with experimental binding affinities of the peptides for PTB domain 1X11, we wanted to investigate if the utility of BT matrix for identification of interaction partners of PTB domains can be validated on a larger dataset. However, for the 1X11 PTB domain no additional experimental data on binding affinity was available. As described in methods section, experimental PTB domain-peptide binding affinity data^11^ was compiled for 10 other PTB domains, where their binding affinity for approximately 123 peptides had been investigated. However, a crucial requirement of our structure based approach is that it should be possible to model the structure of a PTB-peptide complex with reasonable accuracy. Comparison of the sequences of these 10 PTB domains with structures in PDB revealed that, peptide bound structure was available for ShcA (PDB ID: 1SHC). Since ShcA and ShcD domains belong to the same family of proteins and share 73% identity, peptide bound structure could be modeled for ShcD using ShcA-peptide crystal structure as template. When we superimposed 1SHC over 1X11 there were several structural differences and the peptides were superimposing well only in NPxY motif containing region (Fig. 5). 1X11 had a longer α helix in the C-terminus, while the corresponding region was extended coil in 1SHC. During the MD simulation of 1X11 this region remained in α helix conformation throughout the trajectory and never sampled conformations similar to that present in 1SHC. The other noticeable difference was the presence of a longer loop region (loop 3) adjacent to the peptide in 1X11, while corresponding loop was smaller in length in 1SHC. This suggests that 1X11 or any other crystal structure of peptide bound PTB domain cannot be used as templates for modeling peptide bound structures for these 10 PTB domains for which binding affinity data was available. Therefore, we used experimental binding data for only these two PTB domains i.e. ShcA and ShcD in our benchmarking study for pair potential based prediction. Since 1SHC structure was available with phosphotyrosine residue containing peptide, we carried out predictions only for the phosphotyrosine containing peptides interacting with PTB domains ShcA and ShcD. The interaction data for ShcA consisted of 123 substrate peptides (69 binder and 54 non-binder peptides), while the interaction data for ShcD also consisted of 123 substrate peptides (73 binder and 50 non-binder peptides).

**Figure 6:**
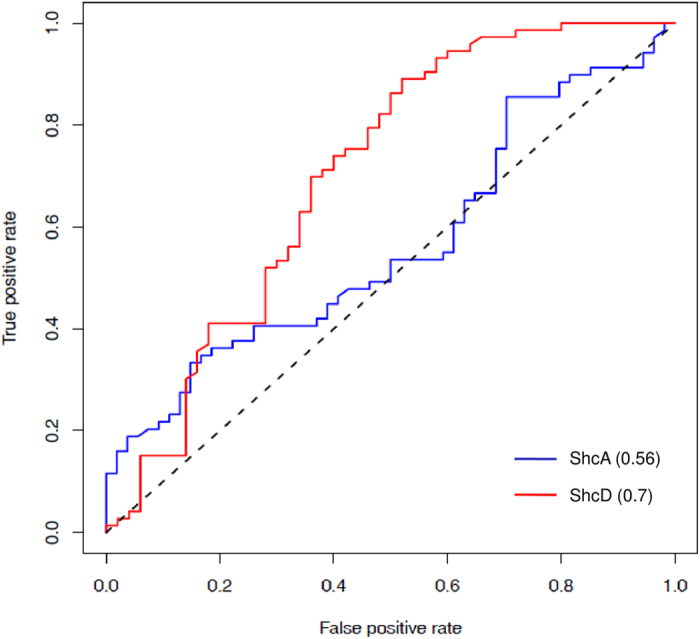
ROC curve for identification of peptide binding partners of ShcA and ShcD PTB domain. ROC curve for 123 PTB-peptide pairs for ShcA is shown in blue color and for the ShcD PTB domain is shown in red color.

In case of ShcA, peptides were modeled into the binding pocket of ShcA by mutating the cognate peptide using SCWRL^26^ (as described in Materials and Methods section) and interaction energy was evaluated using BT matrix. 1SHC structure was available with phosphotyrosine residue containing peptide, therefore ranking was performed only for phosphotyrosine containing peptides. The ranking of peptides was done with phosphorylation mimic where all phosphotyrosine residues in the potential substrate proteins were mutated to glutamate residues. In case of ShcD, the PTB-peptide complexes were modeled using the SWISS-MODEL server and peptide bound crystal structure of ShcA (PDB Id: 1SHC) as the template. The same protocol of ranking the phosphotyrosine peptides as in case of ShcA was followed. The binder and non-binder peptides of ShcA got the binding score in the range of −6.97 to 5.78 where more negative scores indicate more favourable interaction with the PTB domain. The true binder peptides scored between −6.97 to 5.63 and true non-binder peptides had scores in the range −5.06 to 5.78. In case of ShcD domain the scores for true binder peptides ranged from −4.08 to 3.18, while scores for true non-binder peptides were in the range −3.10 to 6.23. The robustness of our protocol for prediction of binding partners was checked by Receiver Operating Characteristic (ROC) curve analysis, which shows true positive rate (TPR) as a function of false positive rate (FPR) by varying the score cut off for predicting a peptide as binder over the entire range. As described in the methods section, ROC analysis was carried out by calculating the number of true positive (TP), false positive (FP), true negative (TN) and false negative (FN) predictions by varying the threshold or cut off score for prediction over the entire range. In fact the score for each peptide in the dataset was used as threshold and TP, FP, TN and FN values were calculated. Each point on the ROC curve corresponds to calculation of TPR and FPR for a given score cut off or threshold. Generally a convex shape of the ROC curve indicate higher TPR at the expense of relatively lower FPR and area under the ROC curve (AUC) is an indicator of predictive power of the computational model. Figure 6 shows the ROC plot for the predictions of 73 binders and 50 non-binders for ShcD PTB domain. Interestingly a good AUC value of 0.7 was obtained for ShcD (Fig. 6, red color) as positive and negative set peptides had overlap in their scores in the range −3.10 to +3.19. On the other hand, the AUC value for ShcA is 0.56 (Fig. 6, blue color) for 123 substrate peptides (69 binder and 54 non-binder peptides), because of larger overlap (−5.06 to 5.63) in the scores of the true binder and non-binder peptides. Thus benchmarking of statistical pair potential based prediction on a larger dataset indicate that, interaction partners of PTB can be predicted with reasonable TPR and FPR values using static crystal structures as template for ShcD, the prediction accuracy is low for ShcA. However, as demonstrated in the current study on 1X11, correlation with experiment can be further improved by incorporating flexibility to the structures with MD simulations. If putative peptide binding residues in the binding pocket can be identified from MD simulations, structure based approach using statistical pair potential can be utilized to screen large number of potential interaction partners of the given PTB domain.

## Discussion

Recent studies on PTB domain containing proteins have revealed that not only NPxY motif but also the residues flanking this motif are crucial determinants of the specificity of the PTB domains towards their substrates^27^. Peptide array results have indicated that peptides containing identical NPXY or NPXpY motifs bind with differential specificity to different PTB domains. Therefore, in this study we have attempted to analyze the sequence and structural features of PTB domains to develop a novel structure based multi-scale approach for deciphering substrate specificity of PTB domains. Analysis of the structures of PTB-peptide complexes in PDB has revealed that despite high divergence in their sequence, PTB domains adopt a conserved structural fold and the substrate peptides bind at the same site in similar conformation and orientation. We have identified seven representative structures of PTB domains, which can be used as structural templates for modeling various PTB-peptide complexes.

In order to explore various conformations in which a peptide can bind to the PTB domain and understand how residues flanking the NPxY motif govern substrate recognition, explicit solvent molecular dynamics simulations have been carried out on six PTB-peptide complexes for which binding affinities were known from experimental studies. Analysis of 100 ns MD trajectories of PTB-peptide complexes has revealed that the core of the PTB domain remains close to starting structure and major conformational fluctuations are restricted to the loop regions. Interestingly, the bound peptide substrate also essentially retained its conformation and orientation in the PTB binding pocket as seen in the crystal structure. The binding free energies for various peptides calculated using MM/PBSA showed good correlation with experimental binding energy with correlation coefficient of 0.63. This suggests that our all atom simulations have modeled the binding modes of various peptides with reasonable accuracy. The binding modes of various peptides could explain how mutation in the regions flanking the NPxY motif alters contacts with the PTB domain and the binding affinity.

Based on analysis of the inter-molecular interactions between the peptide and the PTB domain during the MD simulation, the amino acids lining the binding pockets for various peptide residues were identified. Interestingly, the binding pockets identified from MD simulations had few crucial differences from the binding pockets present in static crystal structures. Our study also demonstrated that based on the binding pockets obtained after MD simulations, the binding energy scores computed using residue based statistical pair potentials also showed good correlation with experimental binding energy. Thus, computational analysis demonstrated the feasibility of multi-scale approach for predicting interaction partners of PTB domains, where in binding pockets can be identified based on detailed all atom simulations and in the next step based on these binding pockets, binding energy scores between PTB domain and peptide can be computed using residue based statistical pair potentials.

We next attempted to benchmark this statistical pair potential based approach on a larger data set comprising of 10 PTB domains and 123 peptides. Out of the 10 PTB domains, crystal structure in complex with peptide was available for only ShcA (PDB ID: 1SHC) and peptide bound structures for another PTB domain ShcD could be modeled using 1SHC because of their close sequence similarity. The set of 123 phosphotyrosine containing substrate peptides were modeled into the binding pocket of ShcA and ShcD PTB domains and the peptides were ranked according to pair potential scores calculated by BT matrix. Interestingly a good AUC value of 0.7 was obtained for ShcD, while AUC for ShcA was 0.56. Even though these results demonstrate the feasibility of using multi-scale approach involving statistical pair potential for identification of interaction partners of PTB domains, further validation on larger number of PTB families will be necessary to increase the robustness of this computational approach. In our earlier work on PDZ domains, we have demonstrated the utility of similar multi-scale approach for PDZ domains^16^. In that study binding pocket residues were identified from MD simulations on a single PDZ domain, but using the same binding pocket pair potential based approach could be successfully used for other families of PDZ domains in mouse genome. However, unlike the PDZ domains which share structure and binding pocket similarity to a much larger extent, in case of PTB domains there are subtle variations in binding pockets of different PTB families due to the involvement of the flexible loop region in interactions with the peptide. Therefore, binding pockets identified from MD simulations on 1X11 could not be utilized for prediction of binding partners for other PTB families. The modest AUC values obtained for pair potential based calculations on static crystal structures indicate that, if representative members from each families are refined by MD simulations, it will be possible to improve predictions of pair potential based approach for PTB domains.

This also highlights some of the limitations of the statistical pair potential based approach. Even though residue based approach is computationally faster, it can be applied only for proteins for which peptide bound structures can be modeled with reasonable accuracy and this method cannot account for allosteric effects. Probably residue based coarse grained models which take into account dynamics of protein-peptide complexes will be able to address these limitations.

## Materials and Methods

### Analysis of three dimensional structures of PTB domains

PTB domain containing structures, both with and without bound substrate peptide were retrieved from the Protein Data Bank (PDB)^17^. It may be noted that search using PDBtools for structures with significant fold level similarity to PTB domain results in 220 structures. However, most of these structures do not belong to PTB family, because proteins like ferm domain and GTPase show fold level similarity to PTB domain. Therefore, structures for PTB domain were obtained by key word search (“PTB” and “PID”) and manual curation. This resulted in a total of 61 structures, consisting of free as well as peptide bound PTB domains. These 61 structures were clustered by applying a cut-off of 90% for sequence identity between any two pairs in the dataset and one structure was picked randomly from each cluster. This resulted in 38 structures and we also included 1X11 to this set as our simulation studies has been carried on 1X11. Thus a set of 39 non-redundant entries out of total 61 structures were taken for further analysis. Structural superposition of these 39 PTB structures with each other was performed using the TM-align program^18^. CLUSTAL Omega^28^ was used for multiple sequence alignment of 39 PTB sequences (after removing redundancy). Phylogenetic tree taking the multiple sequence alignment file as input was built by neighborhood joining method with bootstrapping using PHYLIP package^29^. The phylogenetic tree for these 39 PTB domains showed 7 clusters and one representative member was selected from each cluster randomly. The sequences of each of these 7 structures were pair wise compared with each other using NCBI BLAST program^30^.

### Modeling of PTB-peptide complexes

Since, experimental binding affinity values were available for PTB domain of X11 protein, corresponding native peptide bound (APP) crystal structure (PDB ID: 1X11)^17^ was taken as the template for modeling complexes for all other peptides involving PTB domain of X11. The crystal structure (1X11) of the X11 PTB-peptide complex was available in the dimeric form. However, biologically meaningful oligomerization state of this protein is monomeric. Therefore, only one monomer was used for modeling PTB-peptide complexes. The structure 1X11 lacked coordinates of 43 residues for which no electron density was reported in the crystal structure. The corresponding residues were modeled using automated method of SWISS-MODEL homology modeling server^31^,^32^. The structural models for the remaining PTB-peptide complexes were generated from this model of the native peptide-PTB complex by *in silico* mutation of side chain residues of the bound native peptide QNGYENPTYKFFE using backbone dependent rotamer library approach of SCWRL^26^. The Unbound structure was generated by removing the peptide coordinates from 1X11.

### Molecular dynamics simulations and MM/PBSA

Molecular dynamics (MD) simulations were performed on 6 PTB-peptide complexes and on 1X11 PTB domain alone without the bound peptide using AMBER 12^33^ simulation package and ff03 force field^34^. The complexes were solvated in a rectangular box of TIP3P water molecules^35^. The box extended 8 Å from the outermost protein atoms along x, y and z directions which included around 8000 water molecules. The solvated structures were minimized by steepest descent algorithm using convergence criteria of 0.001 kcal/mole/Å as RMS gradient of potential energy. After minimization, MD simulations were carried out using 2fs time step. The temperature of the system was gradually increased to 300K over a simulation time of 100ps under NVT conditions and Langevin temperature equilibration scheme was used to maintain the temperature. The pressure equilibration to 1 atm using NPT conditions for a further period of 100 ps was done using isotropic position scaling to maintain the pressure with relaxation time of 2 ps. Supplementary Figure S6 shows the variation of box volume as a function of simulation time to indicate equilibration of box volume. After 200 ps of equilibration, production dynamics simulations were carried out under NVT conditions at 300 K. A cut-off of 8 Å was used to compute non-bonded interactions and long-range electrostatic interactions were computed using Particle Mesh Ewald (PME) approach^36^. For each of these 6 PTB-peptide complexes and PTB alone structure, 100 ns trajectories were computed. PTRAJ module of AMBER12 package and Perl scripts developed in house were used for analyzing MD trajectories.

For calculation of the binding free energies from 100 ns explicit solvent MD trajectories for different PTB-peptide complexes, snapshots at an interval of 50 ps were extracted from last 20 ns of each trajectory. MM/PBSA approach with salt concentration of 0.15 M was used for calculating the binding free energy, which uses an implicit solvent model^13^. The free energy was calculated for each molecular species namely, PTB-peptide complex, receptor (PTB domain) and ligand (peptide). The peptide binding free energy was computed as the difference.


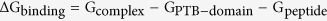


### Binding pocket analysis & calculation of pair potential scores

Last conformation was extracted from 100 ns MD trajectories for a given PTB-peptide complex. In each of these PTB-peptide complexes, the contacting residue pairs between domain and peptide were identified using the criteria of any two atoms of the residue pair being at a distance less than 4.5 Å to construct binding pockets for each peptide residue. Based on the contacting residue pairs between protein and peptide, the binding energy was evaluated using Betancourt-Thirumalai (BT)^24^ statistical pair potential matrix. The values have been derived from the observed frequency of contacts between various amino acid pairs in a non-redundant set of proteins corresponding to different folds. Hence, statistical pair potential used for scoring is not specific for PTB-peptide complexes under study.

### Benchmarking of the predictive power of statistical pair potential on a larger dataset

We also attempted to investigate, whether the binding pocket residues identified from modeled PTB-peptide structures can be suitably used for predicting the interaction partners by using statistical pair potentials directly without performing all-atom simulations. In order to benchmark this multi-scale approach, peptide binding data for 10 PTB domains reported by Smith *et al*.^11^ was compiled and utilized. Peptide array approach had been used by Smith *et al*. to screen a library of human proteome derived NXXY sequence in both phosphorylated and non-phosphorylated forms to explore substrate specificity of 10 PTB domains. In this study densitometry measurements had been used to categorize binding reactions as strong, intermediate, weak and undetectable in terms of a color gradient. We used this data to derive experimentally determined specificity for 10 PTB domains for 123 12-mer peptides containing NXXY motifs as well as their phosphorylated counterparts in a binary form as binders and non-binders. Strong binding reactions were classified as binders while others were termed non-binders. This provided us experimental binder vs non-binder data for 10 PTB domains, which we wanted to use for benchmarking the predictive power of our pair potential based approach.

We took sequences of those 10 PTB domains and carried out BLAST search against PDB database. Crystal structure of only ShcA PTB domain was found in complex with peptide having phosphorylated tyrosine residue (PDB ID: 1SHC). Since the crystal structure used as template had a peptide bound to PTB domain, while modeling each of the 123 peptides (69 binder and 54 non-binder peptides) the backbone conformation of the modeled peptide was kept same as that in the structural template and the side chains were only replaced using SCWRL program^26^. The peptide sequences were aligned to the sequences of the crystal structure bound peptide using NXXY motif as reference. The peptide bound structure for ShcD was modeled using 1SHC structure as template in the SWISS-MODEL^31^,^32^ server and 123 substrate peptides (73 binder and 50 non-binder peptides) were modeled in the binding pocket of ShcD using SCWRL. To mimic tyrosine residue phosphorylation, all tyrosine residues in the substrate sequences were mutated to glutamate. The interaction energy of every PTB-peptide complex was then calculated using Betancourt-Thirumalai (BT) residue based statistical pair potential^24^.

Based on this computational binding energy, the peptide was ranked as a binder if its score was lower than a given score cut off and non-binder otherwise. If the corresponding peptide was also a binder as per experimental data, the prediction was called true positive (TP). If it was non-binder in the experimental data the prediction was ranked as false positive (FP). Similarly, if the experimentally known binder peptides had scores above cut off, they were counted as false negative (FN) and true negative (TN) otherwise. For each value of the score cut off TPR and FPR values were computed from TP, FP, TN and FN values. TP, FP, TN and FN values and consequently TPR and FPR were recomputed by varying the score cut off over the entire range of the pair potential score. ROC curve was generated by plotting TPR as a function of FPR for different values of score cut off. Depending on their experimental label (positive or negative) from Smith *et al*.^11^, they were classified as true positive (TP), true negative (TN), false positive (FP) and false negative (FN). Area under the curve (AUC) was calculated in ROC plot to check the statistical significance of the protocol. In ROC curve, TPR (true positive rate) is plotted against the FPR (false positive rate) where TPR is the fraction of true positives among all positives (TPR = TP/(TP + FN)) and FPR is the fraction of false positives among all negatives (FPR = FP/(FP + TN)).

## Additional Information

**How to cite this article**: Sain, N. *et al*. Understanding the molecular basis of substrate binding specificity of PTB domains. *Sci. Rep.* **6**, 31418; doi: 10.1038/srep31418 (2016).

## Supplementary Material

Supplementary Information

## Figures and Tables

**Table 1:** List of peptides that were modeled in complex with PTB domain 1X11, their K_d_ values, experimental binding free energy and free energies computed by MM/PBSA and BT pair potential matrix.

Protein 1X11	Relative Affinity	ΔG = RTlnK_d_ (kcal/mol)	PBTOT (kcal/mol)	PP score (BT matrix)
QNGYENPTYKFFE	1	−8.906	−80.42 (8.87)	−6.05
QNGYANPTYKFFE	0.19	−7.901	−75.11 (8.73)	−3.08
QNGYENPTYKAFE	0.1	−7.491	−76.47 (7.42)	−0.87
QNGYENPTYKFAE	0.11	−7.551	−69.06 (10.15)	−2.51
QNGYENPTYKFFA	0.56	−8.541	−71.91 (9.10)	−3.25
GYENPTYKFF	0.07	−7.324	−58.32 (5.88)	−2.43
Corr. Coeff.	—	—	0.63	0.85

MM/PBSA energy (PBTOT) values are averages values over the last 20 ns of the 100 ns MD trajectory and the numbers in parentheses indicate standard deviations. Pair potential score (PP score) has been computed for the final structures obtained from the MD simulation. The last two columns in the last row show the correlation coefficient between experimental and computed binding free energy values. p-value for correlation coefficient between experimental and pair potential score is found to be 0.03.
