# Supplementary material for: Understanding the molecular basis of substrate binding specificity of PTB domains
Source: Sci Rep. 2016 Aug 16;6:31418. doi: 10.1038/srep31418 (PMC4985636; doi:10.1038/srep31418)
Supplement: Supplementary Information [file srep31418-s1.doc]

**Supplementary Information**

**Understanding the molecular basis of substrate binding specificity of PTB domains**

**Neetu Sain**^1,#^**, Garima Tiwari**^1,2,#^ **and Debasisa Mohanty**^1^**^*^**

^1^Bioinformatics Center, National Institute of Immunology, Aruna Asaf Ali Marg,

New Delhi – 110067, India.

^2^Current address: Biomolecular Modeling and Design Division, Bioinformatics Institute, A*STAR (Agency for Science, Technology and Research), 30 Biopolis Street, #07-01 Matrix, Singapore 138671.

^#^Both authors have equal contribution.

# ^*^Correspondence

Debasisa Mohanty, Bioinformatics Center, National Institute of Immunology,

Aruna Asaf Ali Marg, New Delhi – 110067, India.

# E-mail: [deb@nii.res.in](mailto:deb@nii.res.in); [deb@nii.ac.in](mailto:deb@nii.ac.in)

# Phone : +91 11-26703749

**E-mail address of the author**

Neetu Sain: [neetu.sain@nii.ac.in](mailto:neetu.sain@nii.ac.in)

Garima Tiwari: [garimat@bii.a-star.edu.sg](mailto:garimat@bii.a-star.edu.sg)

**Supplementary Table S1.** List of peptides with known binding affinity [^1^](#_ENREF_1) which have been modeled in complex with PTB domain 1X11 and details of MD simulations carried out on each PTB-peptide complex.

| **Peptide** | **Sequence** | **Simulation length** |
| --- | --- | --- |
| **13mer** | **QNGYENPTYKFFE** | **100ns** |
| **E5A** | **QNGYANPTYKFFE** | **100ns** |
| **F11A** | **QNGYENPTYKAFE** | **100ns** |
| **F12A** | **QNGYENPTYKFAE** | **100ns** |
| **E13A** | **QNGYENPTYKFFA** | **100ns** |
| **10mer** | **GYENPTYKFF** | **100ns** |

**Supplementary Table S2.** List of residues of the PTB domain, which are in contact with the residues of the peptide after 100ns MD simulations in native peptide complex. Nature of contacts are represented as BB for backbone-backbone, BS for backbone-sidechain, SB for sidechain-backbone and SS for sidechain-sidechain interactions.

| **Peptide Residue** | **Protein Residue** | **Residue Number** | **Nature of contacts** |
| --- | --- | --- | --- |
| **GLN(Q)** | **MET (M)** | **354** | **BS** |
|  | **ALA (A)** | **420** | **BS** |
|  | **ASP (D)** | **421** | **BB BS SB SS** |
|  | **GLN (Q)** | **469** | **SS** |
| **ASN (N)** | **TYR (Y)** | **418** | **SS** |
| **GLY (G)** | **TYR (Y)** | **418** | **BS SS** |
|  | **ILE (I)** | **419** | **BB** |
|  | **ALA (A)** | **420** | **BS** |
| **TYR (Y)** | **TYR (Y)** | **418** | **BS BB** |
|  | **ILE (I)** | **419** | **BB BS SS** |
|  | **GLY (G)** | **476** | **SB** |
|  | **PHE (F)** | **479** | **SS SB** |
|  | **SER (S)** | **480** | **SS SB** |
|  | **TYR (Y)** | **483** | **SS** |
| **GLU (E)** | **ARG (R)** | **353** | **SS** |
|  | **SER (S)** | **417** | **BB SB** |
|  | **TYR (Y)** | **418** | **BS** |
| **ASN (N)** | **LEU (L)** | **413** | **SB** |
|  | **ARG (R)** | **498** | **SB** |
|  | **ILE (I)** | **416** | **SB** |
|  | **SER (S)** | **417** | **BB SB** |
|  | **PHE (F)** | **479** | **BS SS** |
| **PRO (P)** | **PHE (F)** | **479** | **SB SS** |
|  | **TYR (Y)** | **483** | **SS** |
| **THR (T)** | **ARG (R)** | **414** | **BS SB SS** |
|  | **PHR (F)** | **479** | **SS** |
|  | **ALA (A)** | **482** | **SS** |
| **TYR (Y)** | **ARG (R)** | **414** | **BS SB SS** |
|  | **ILE (I)** | **416** | **SB** |
|  | **SER (S)** | **417** | **SB SS** |
|  | **ARG (R)** | **431** | **SS** |
| **PHE (F)** | **TYR (Y)** | **483** | **SS** |
|  | **PHE (F)** | **486** | **SS** |
| **PHE (F)** | **ARG (R)** | **117** | **BS SS** |
|  | **ALA (A)** | **482** | **SB** |
|  | **GLU (E)** | **485** | **SB SS** |
|  | **PHE (F)** | **486** | **SB SS** |
| **GLU (E)** | **ARG (R)** | **117** | **BS SS** |
|  | **ARG (R)** | **431** | **SS** |
|  | **ARG (R)** | **433** | **SS** |

**Supplementary Table S3.** List of residues of the PTB domain, which are in contact with the mutated residues of the peptides after 100ns MD simulations for four mutant complexes as compared to native peptide complex binding pocket residues. The corresponding binding energy score calculated using BT matrix is written in parentheses.

| **Binding pocket** | **1X11 WT QNGYENPTYKFFE** | **1X11mpepE5A** | **1X11mpepF11A** | **1X11mpepF12A** | **1X11mpepE13A** |
| --- | --- | --- | --- | --- | --- |
| **Pocket5 (E to A)** | **Y418 S417 R353**  **(-0.81)** | **S417 Y418 F479 (-0.33)** | **-** | **-** | **-** |
| **Pocket11 (F to A)** | **F486 Y483**  **(-1.31)** | **-** | **R433 F486 Y483 (-0.21)** | **-** | **-** |
| **Pocket12 (F to A)** | **R414 A482 F486 E485 (-0.73)** | **-** | **-** | **R414 R431 (0.54)** | **-** |
| **Pocket13 (E to A)** | **R414 R431 R433**  **(-2.25)** | **-** | **-** | **-** | **R414 E444 R436 N442 V146 (0.83)** |

**
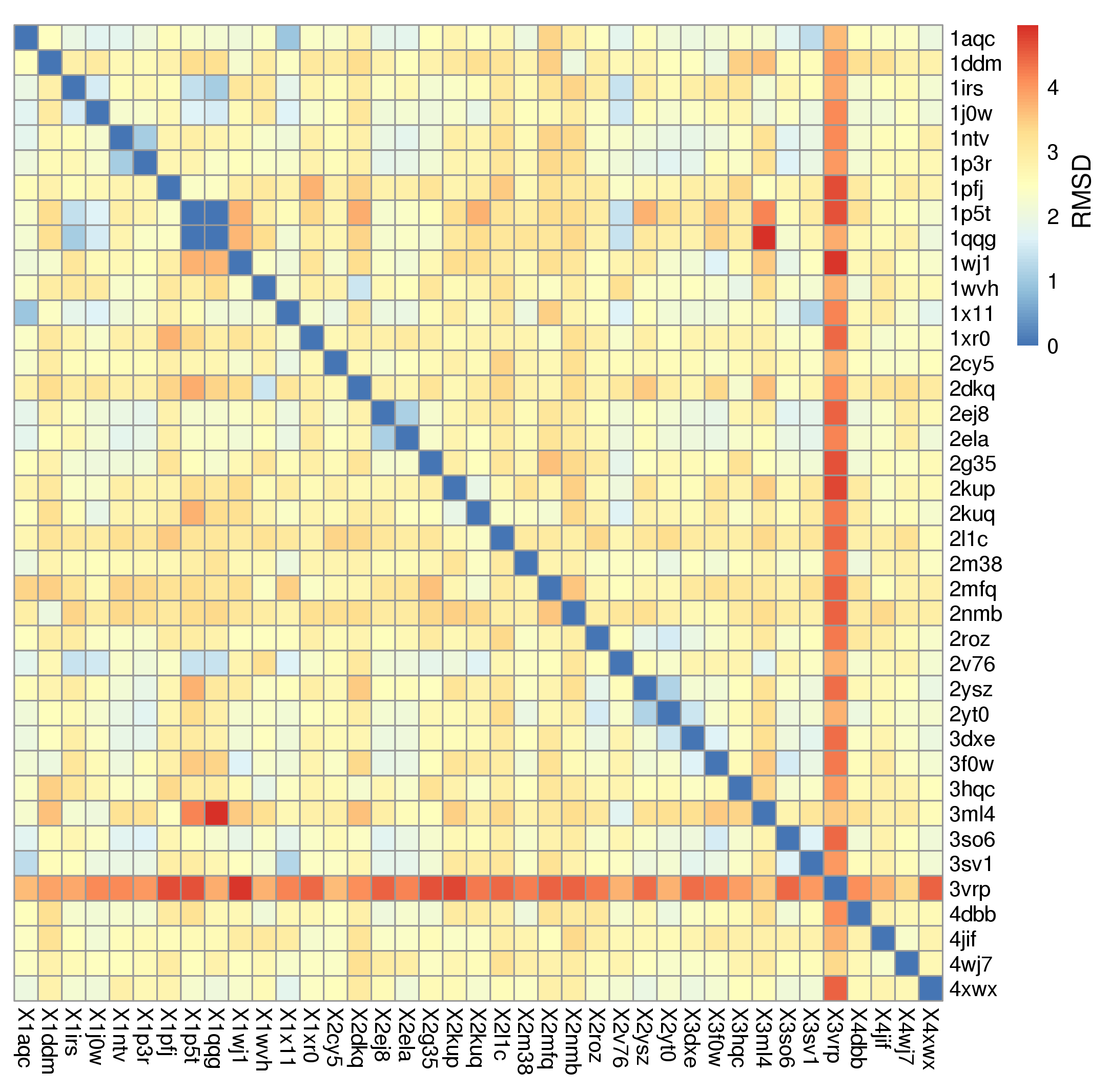
Supplementary Figure S1.** Pair wise structural comparison between 39 PTB domain structures obtained after clustering of 61 PTB retrieved from PDB using a sequence identity cut off of 90%. The list of 61 PDB IDs retrieved from PTB based on key word search are

1J0W, 4XWX, 2EJ8, 2GJY, 2ELA, 1X11, 2DKQ, 1N3H, 1OY2, 2MFQ, 4JIF, 2V76, 1WVH, 2LOZ, 2L1C, 1XR0, 1DDM, 2NMB, 1AQC, 1NTV, 1IRS, 1QQG, 1NU2, 2M38, 2CY4, 2CY5, 4WJ7, 2G35, 1P5T, 4DBB, 1UEF, 3SO6, 3ML4, 2KUP, 1P3R, 1PFJ, 2KUQ, 1OQN, 1SHC, 2YS5, 1M7E, 2YT2, 2DYQ, 3HQC, 4DX8, 1WJ1, 4DX9, 3SV1, 3VRN, 3DXC, 3DXD, 3DXE, 3VRO, 3VRP, 3VRQ, 3VRR, 2ROZ, 2F0W, 2YSZ, 2YT0, 2YT1

**
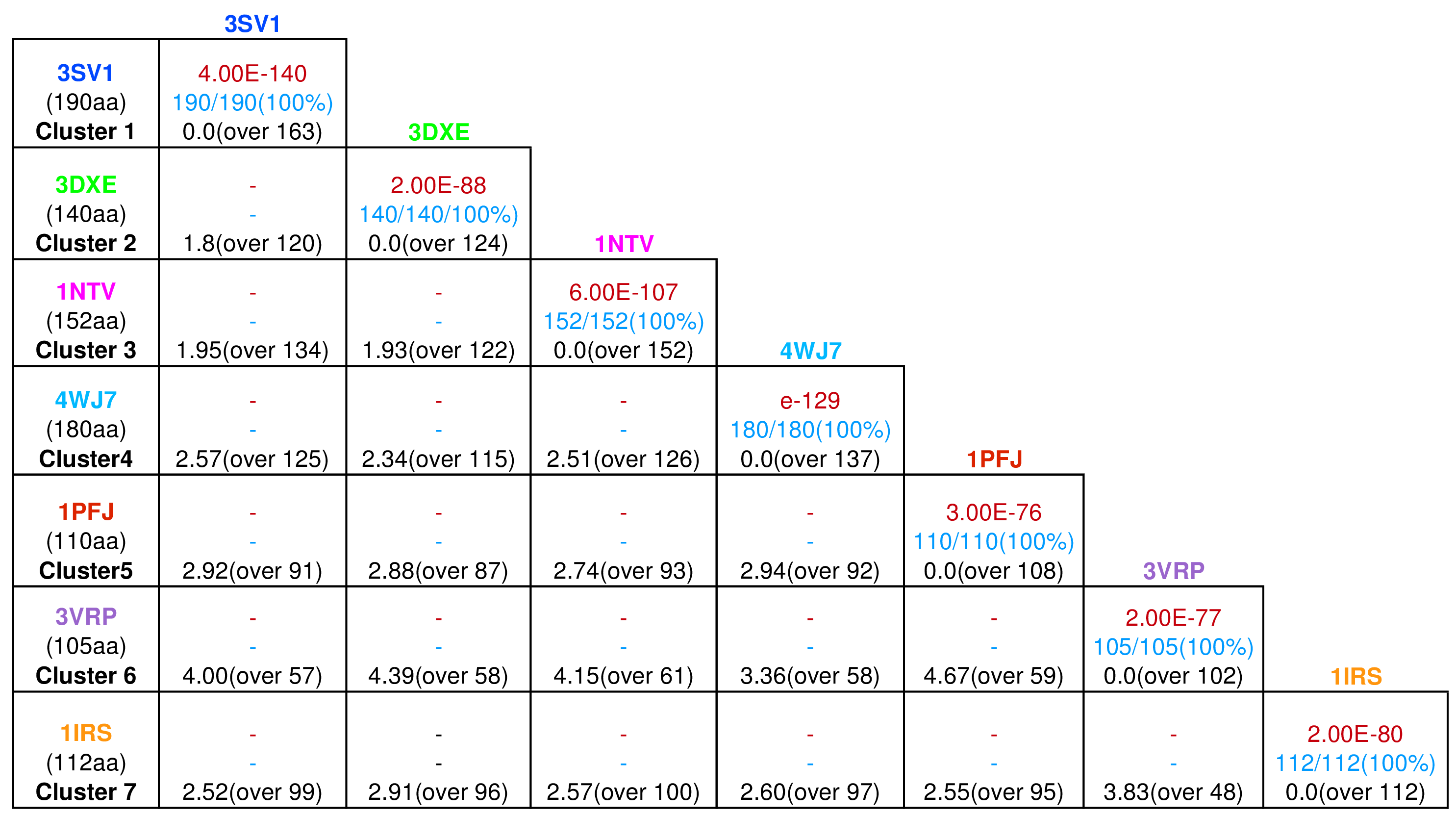
**

**Supplementary Figure S2.** Pair wise sequence and structural comparison between seven representative structures from seven different clusters of PTB domains. First column indicate PDB IDs of seven representative structures with the number of residues in parenthesis. The table indicates e-value (red colored), percentage similarity (cyan colored) and RMSD (black colored) and the number of aligned residues in the structural alignment (in parentheses). E-values and percentage similarities are not shown for all as no statistically significant sequence alignment could be obtained.

**
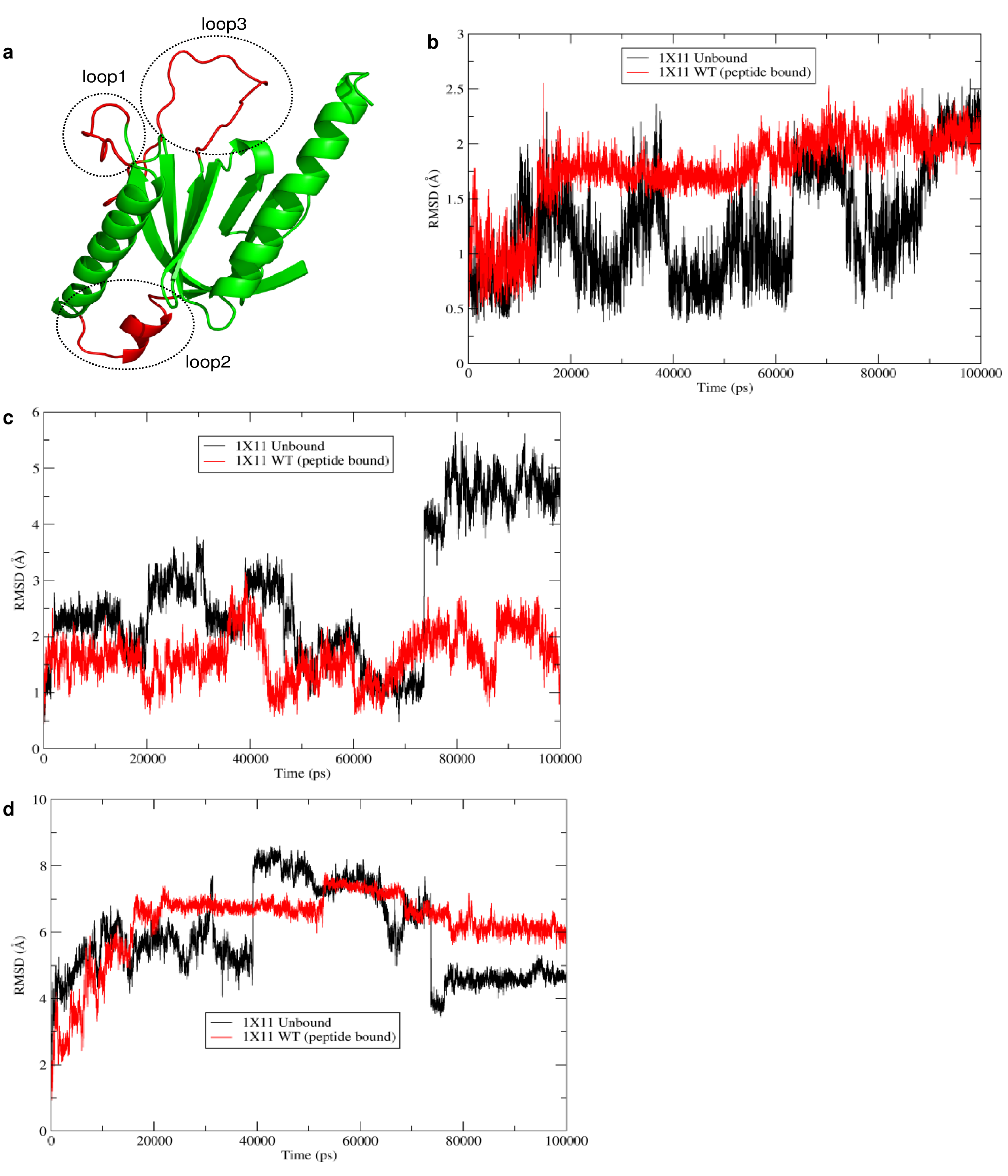
**

**Supplementary Figure S3.** Comparison of loop flexibilities in the unbound versus native peptide bound structure of 1X11 PTB domain. a) All three loops of 1X11 PTB domain are represented as loop1, loop2 and loop3 b) RMSD plot for loop1. c) RMSD plot for loop2. d) RMSD plot for loop3.


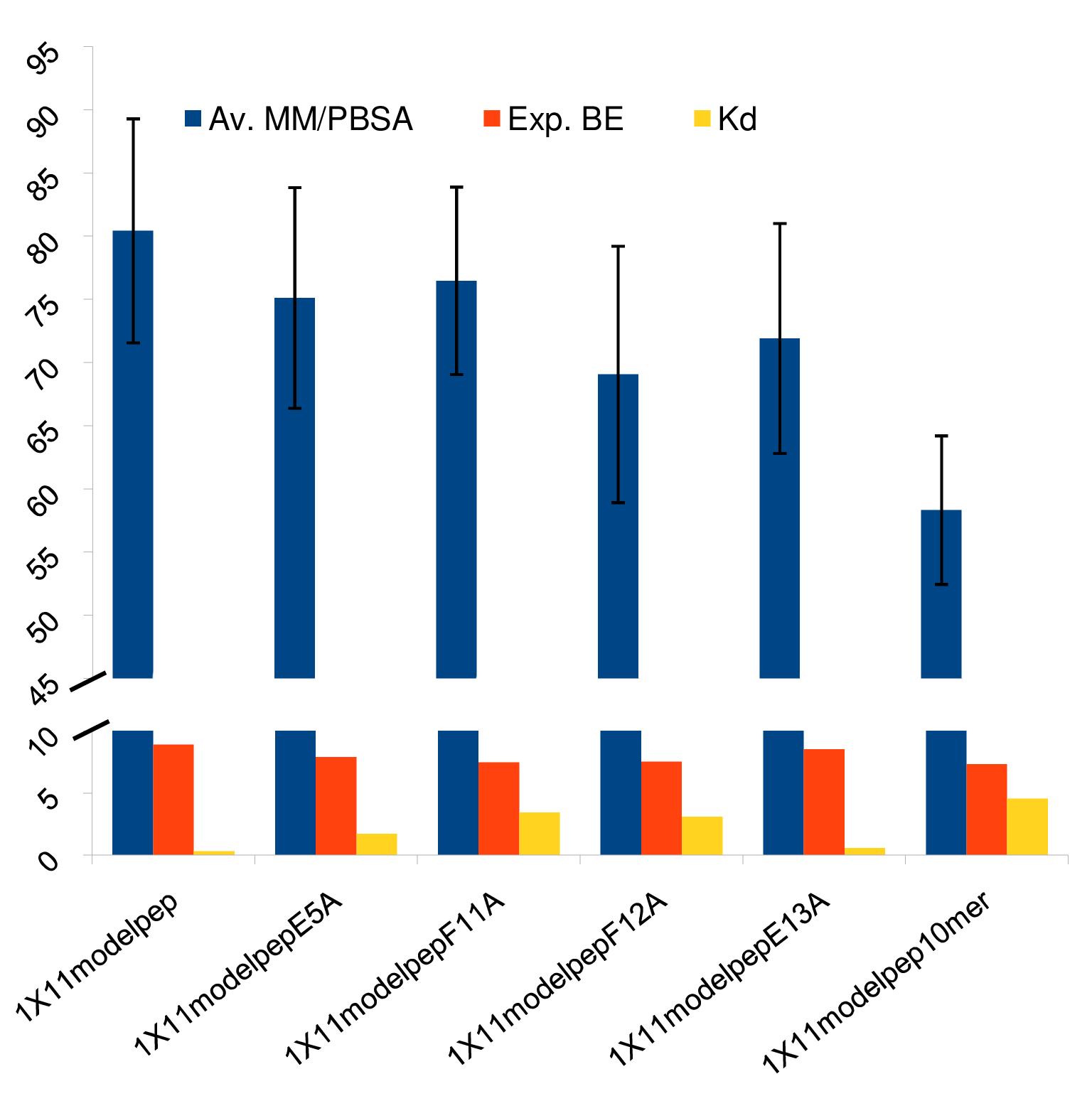


**Supplementary Figure S4.** Bar graph showing binding energy values calculated from MM/PBSA (for the sake of simplicity the values are plotted on +ve Y-axis), experimental binding energy values calculated from the Kd values for all 6 PTB peptide complexes. The error bars on binding energy values are standard deviations with respect to the average MM/PBSA energy values computed from last 20ns of the 100ns MD trajectories.


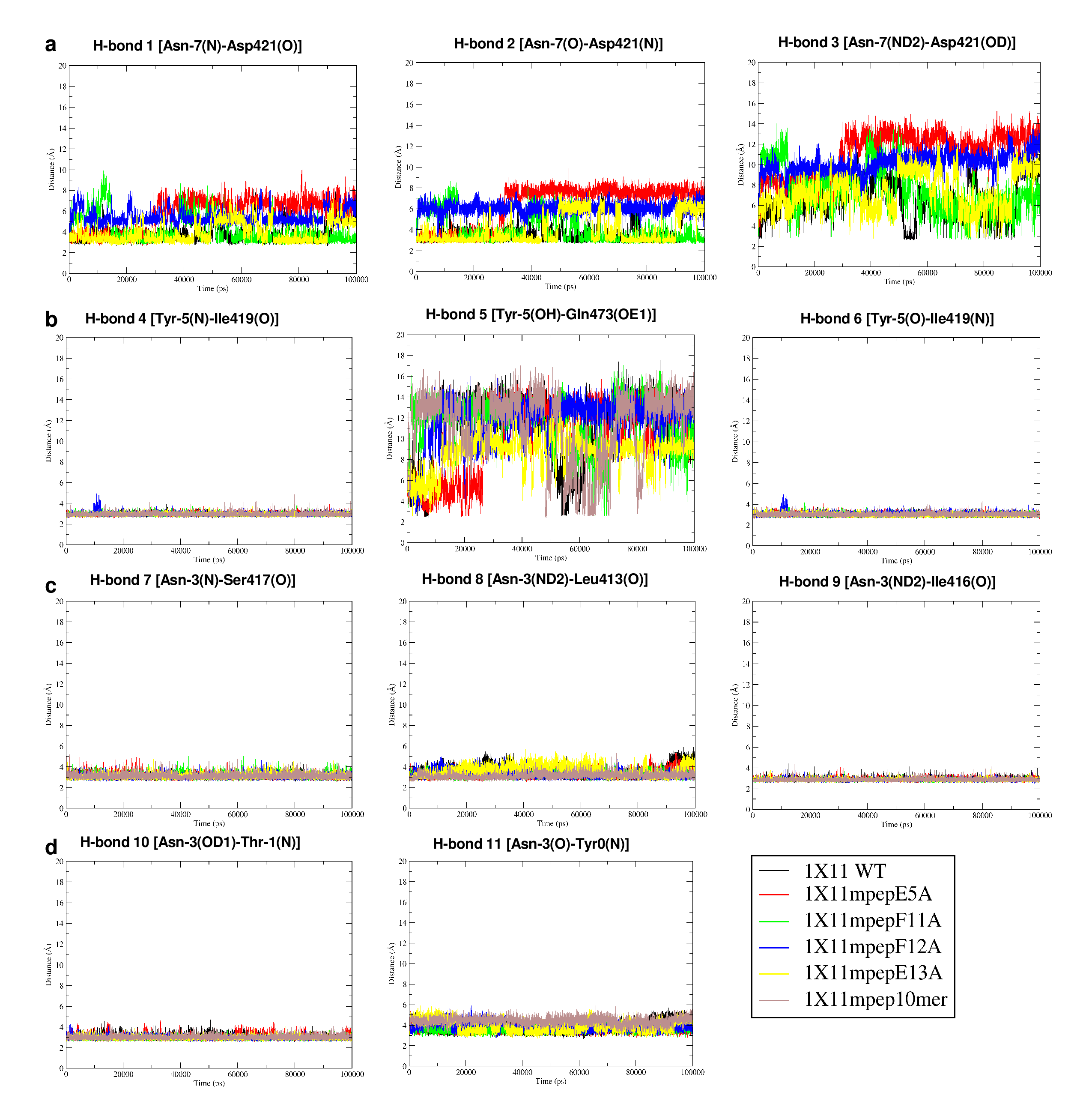


**Supplementary Figure S5.** (a) Hydrogen bond between Asn (-7) of APP peptide and Asp 421 of X11 PTB domain numbered as H-bond 1, 2 and 3, where H-bond 1 and 2 represents the backbone hydrogen bonding and H-bond 3 represents the side chain-side chain hydrogen bond. (b) H-bond 4 and 6 depict the backbone hydrogen bonding between Tyr (-5) of APP peptide and Ile 419 of X11 PTB domain. H-bond 5 involves side chain OH of Tyr residue and OE1 of Gln 473. (c) Hydrogen bond between backbone atoms of Asn (-3) of APP peptide and Ser 417 of X11 PTB domain is numbered as H-bond 7. H-bond 8 and 9 involves the side chain nitrogen of Asn (-3) and backbone O of Leu 413 and Ile 416 respectively. (d) Hydrogen bond numbered as H-bond 10 is between side chain oxygen of Asn (-3) and backbone nitrogen of Thr (-1) of APP peptide itself. H-bond 11 is again between the peptide residues involving backbone atoms of Asn (-3) and Tyr 0.

**
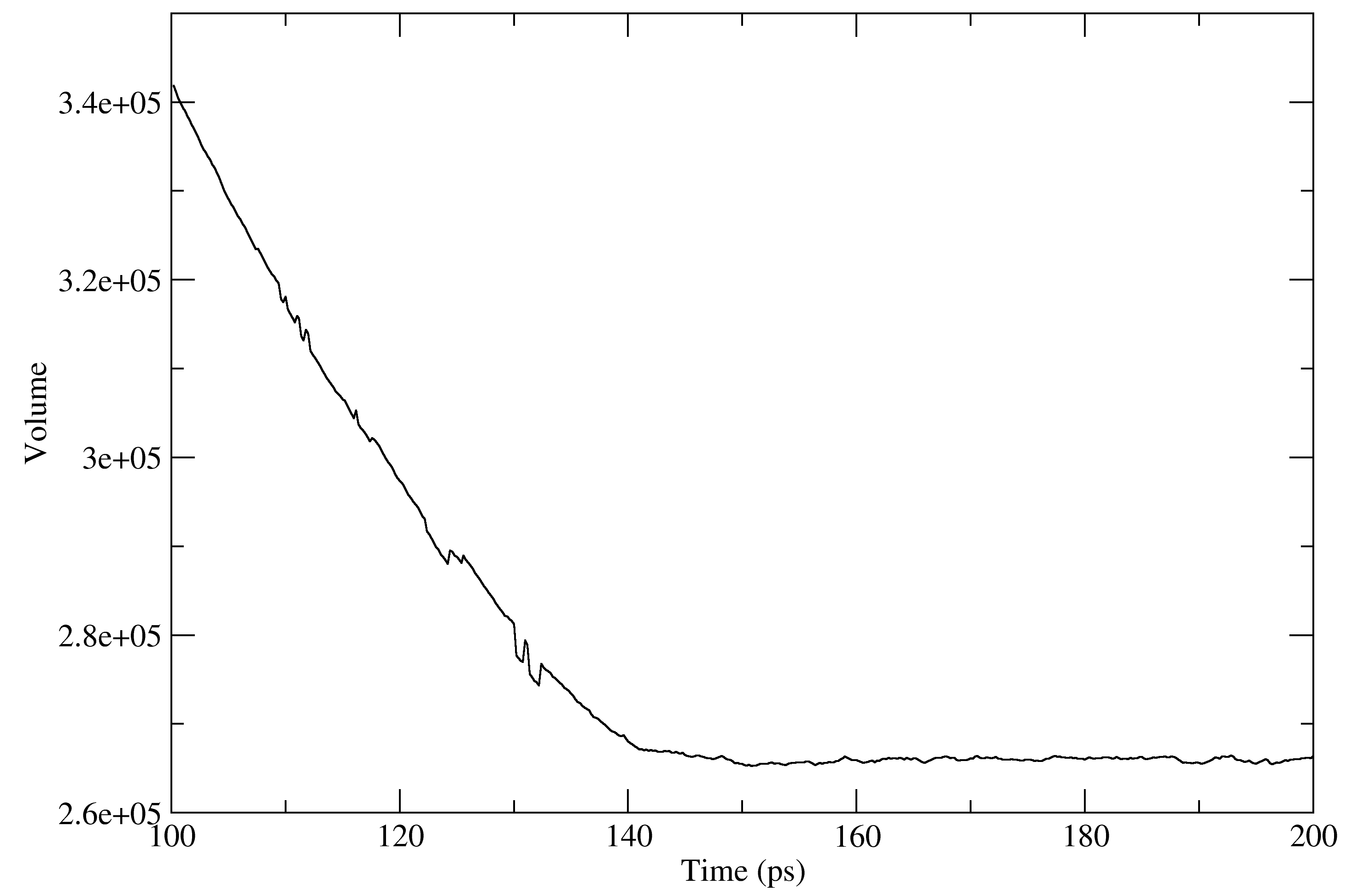
**

**Supplementary Figure S6.** Volume (Å^3^) versus Time (ps) plot showing fluctuations in volume of the system during NPT run. Volume of the system is equilibrated after 40ps.

**Supplementary References**

1 Zhang, Z. *et al.* Sequence-specific recognition of the internalization motif of the Alzheimer's amyloid precursor protein by the X11 PTB domain. *Embo J* **16**, 6141-6150 (1997).
